# Adhesions and Anti-Adhesion Systems Highlights

**Published:** 2019-06

**Authors:** LA Torres-De La Roche, R Campo, R Devassy, A Di Spiezio Sardo, A Hooker, P Koninckx, B Urman, M Wallwiener, RL De Wilde

**Affiliations:** University Hospital for Gynecology, Pius Hospital, University Medicine Oldenburg, Carl von Ossietzky University Oldenburg, 26121, Germany;; Leuven Institute for Fertility and Embryology, Tiensevest, 3000, Leuven;; Dubai London Clinic and Speciality Hospital, Dubai, 3371500, UAE;; University of Naples Federico II, Napoli, 80131, Italy;; Zaans Medical Centre, Zaandam, 1502, Netherlands;; UZ Leuven Campus Gasthuisberg, Leuven, 3000, Belgium;; Koç University Faculty of Medicine, Department of Obstetrics and Gynecology, Istanbul, Turkey, 34330, Turkey;; Heidelberg University Women's Hospital, Heidelberg, 69115, Germany.

**Keywords:** Peritoneal adhesions, prevention, antiadhesion agents, gynecological surgery

## Abstract

The peritoneal and intrauterine cavities are lined by fragile membranes with a high-wound healing capacity, e.g. repairing the endometrium in its cyclical “injury and scar-free repair process” during menstruation. However, peritoneal and intrauterine fibrosis and adhesions can develop after surgical trauma through activation of molecular, immune and genetic mechanisms. During procedures with a high-risk of adhesions, the use of new peritoneal and intrauterine conditions in combination with anti-adhesion substances are promising measures to preserve peritoneal and endometrial function and avoid the most common complication of gynecological surgery. Highlights of adhesions and anti-adhesion prevention techniques in laparoscopic, laparotomic and hysteroscopic surgeries are discussed in this paper. Unfortunately, evidence is lacking to prove the superiority of one technique over its counterparts in terms of postoperative adhesions, such as instrumentation, type of energy, distending media, and intracavitary pressure. Additionally, there is limited evidence about the efficacy and outcomes of techniques and adjuvant measures used during adhesiolysis. The definition of a universal intrauterine adhesions classification scheme as well as a prognostic scoring system to identify women at high risk of postoperative adhesions are necessary for advising those who could benefit the most of the use of antiadhesion barriers.

## Introduction

Adhesions are the most frequent complication of surgery. In abdominal operations, they can provoke severe problems such as chronic pain, infertility, and even bowel obstruction. Standard of care to avoid them is a qualified surgical technique, reducing tissue and, especially, peritoneal trauma. In the future, adhesion-prophylactic agents and adhesion-reducing measures will gain more importance. In order to further optimize adhesion prophylaxis, continuous research is necessary.

This manuscript expands the level of knowledge in this complicated field. In particular, evidence from new laparoscopic and hysteroscopic approaches, including peritoneal conditioning, hysteroscopic morcellation, and the application of barrier agents for intrauterine adhesion prophylaxis, are presented in detail. Furthermore, the necessity for a unique classification of intrauterine adhesions and a prognostic score system is highlighted. The presentation of this information should serve to enhance awareness and the clinical science surrounding intrauterine adhesions and their potential consequences, giving benefits to patients, surgeons and nurses, as well as to governments and health insurance companies by reducing adhesion-related diseases and costs.

Abdominal and intrauterine postsurgical adhesions seem to be an issue of relatively low concern among gynecological surgeons, as few have been found to be sufficiently aware of adhesion-related issues and their consequences for their daily practice (Yu et al., 2008; Schreinemacher et al., 2010; Wallwiener et al., 2013). Available evidence shows a high level of adhesiogenesis associated with routine abdominal and intrauterine procedures, even though the development of adhesions is not fully understood (De Wilde et al., 2016). Moreover, there is a lack of evidence regarding the effects of different hysteroscopic techniques and antiadhesion agents on postsurgical intrauterine adhesions (IUA), which are often silent and have a potential to negatively impact fertility (Gambadauro et al., 2012). Highlights of adhesions and antiadhesion prevention techniques in laparoscopic, laparotomic and hysteroscopic surgeries are discussed in this paper.

The peritoneal cavity consists of two thin membranes (40 μm) of tortuous overlapped mesothelial cells (visceral and parietal peritoneum) (Buţureanu and Buţureanu, 2014; Isaza-Restrepo et al., 2018). These cells lie tightly close to each other, have numerous apical microvilli and intercellular junctions with liquid-filled space in-between. They rest on a basal lamina of connective tissue comprised of collagen, mucopolysaccharides, parenchymal cells, fibroblasts, adipocytes, pain receptors, and blood and lymph vessels. This membrane has many functions and capacities including selective cell transport; immune induction, modulation, and inhibition; tissue repair and scarring; adhesion and tumoral dissemination prevention; epithelial-mesenchymal transition, and long-term diffusive or convective fluid transport (ultrafiltration). Because of its structure and functions, the peritoneal membrane could be considered an organ and not simply as a serous membrane acting only as a physiological protective barrier of the gut and abdominopelvic organs. When peritoneum is exposed to surgical trauma, dialysis substances or infectious injuries, multiple pro-inflammatory and gene expression mechanisms are activated that can lead to peritoneal fibrosis and adhesions (Saxena, 2008; Bellón et al., 2011)

The intrauterine cavity is covered by a double-endometrial cell layer, basal and functional, that acts as a highly sensible and complex multicellular structure, involving interactions of hormonal, immune, endocrine and vascular systems (Liu et al., 2019). It undergoes a cyclical “injury and scar-free” repair process during menstruation, in a similar way to classic wound healing, including inflammation, angiogenesis, tissue formation, and tissue remodeling (Maybin and Critchley, 2015). Nevertheless, fibrosis and IUA can develop if disruption of the basalis layer occurs during intrauterine operations. Several mechanisms have been postulated to be involved in the physiopathology of IUA, including hypoxic injury, inflammation, decreased angiogenesis, and immune and molecular mechanisms. A reverse process of epithelial-to-mesenchymal transition (EMT), aberrant myofibroblast differentiation, bizarre stem cells reparation, and impaired endometrial growth have also been elucidated (Maybin and Critchley, 2015; Liu et al., 2019). When adhesions are formed, the functional layer is replaced by an inactive epithelial monolayer that is much less or nonresponsive at all to hormone stimulation (Metello and Jimenez, 2018).

Postoperative IUA represent an important clinical issue because they may result in infertility, recurrent miscarriages, irregular cycles with dysmenorrhea, and pelvic pain. Nonetheless, there is a lack of evidence to prove the superiority of one technique over its counterparts in terms of preventing postoperative IUA, including instrumentation, type of energy, distending media and intrauterine pressure.

## Peritoneal conditioning: What’s in a word?

### Risks factors

A surgical trauma results within a few minutes in exudation, and platelet and fibrin deposition. After several hours, the denuded area is covered by tissue repair cells, including macrophages, generating a cascade of events. Epithelial repair starts on the first day and is terminated by the third day. If repair is delayed by decreased fibrinolysis, local inflammation, or factors in peritoneal fluid, fibroblast growth starts on the third day and angiogenesis on the fifth day, resulting in adhesion formation.

For this mechanism, quantitatively more important are factors released into the peritoneal fluid after retraction of the fragile mesothelial cells and acute inflammation of the entire peritoneal cavity (Koninckx et al., 2016). This is caused by mechanical trauma, hypoxia and reactive oxygen species resulting from open surgery, desiccation, or presence of blood. The severity increases with higher temperatures.

The repair can be delayed by factors that maintain local inflammation, such as necrotic tissue, sutures, hypoxia, oxidative stress, and infection. Even more important are factors derived from retraction and bulging of mesothelial cells and acute inflammation in the entire peritoneal cavity.

### Protective factors

Protection against adhesion formation lies in the prevention of acute inflammation in the peritoneal cavity by means of gentle tissue handling; the addition of more than 5% N_2_O to the CO_2_ pneumoperitoneum; cooling the abdomen to 30°C; anti-desiccation measures [heated-humidified gases like CO_2_, N_2_O or He]; a short duration of surgery; and finally, meticulous hemostasis, thorough lavage, application of a barrier to injury sites, and administration of dexamethasone (Koninckx et al., 2016).

### Proof of concept

The proof of concept was provided by a translational research evaluation, analyzing the efficacy of full-conditioning (FC) to prevent adhesions (Koninckx et al., 2013). The conditioning consisted of decreasing acute inflammation by combining 86% CO_2_, 10% N_2_O and 4% O_2_ in the pneumoperitoneum, cooling of the peritoneal cavity, humidification, heparinized rinsing solution, and 5 mg of dexamethasone as demonstrated in animal models. A randomized controlled trial compared standard laparoscopy with FC, together with an auto-crosslinked polysaccharide (ACP) hyaluronic acid gel barrier (in a 2:3 ratio) in 44 women undergoing deep endometriosis surgery. The results showed that: FC + ACP hyaluronic acid gel barrier prevented adhesions in 75% of cases whereas all women had adhesions in the control group (p < 0.005); density and severity of adhesions were less (p < 0.001) and, in the FC + ACP hyaluronic acid gel barrier group, CO_2_ resorption, postoperative pain, and C-reactive protein (CRP) concentrations were lower, while clinical recovery was faster and time to first flatus shorter. It is expected that FC will also reduce the postoperative fatigue syndrome occurring in some 30% of women (Koninckx et al., 2016).

## Prognostic classification and standardized treatment of intrauterine adhesions

### 

The occurrence of IUA following surgery is the result of various conditions and leads to different symptoms. Thereby, in the literature, the prevalence varies according to conditions and procedures (Table I), such as dilatation and curettage (up to 67%), endometrial ablation (35%), submucosal myomectomy (31%), and bilateral uterine artery embolization (14%); being uncommon after septum (6.7%) and polyp resection (1,6%). Postabortion, postpartum infections and other infections (tuberculosis, schistosomiasis) are less related to IUA (4%) (Yu et al., 2008; Metello and Jimenez, 2018) (Table I).

**Table I:** — Occurrence of intrauterine adhesions following surgery according to conditions and procedures.

Author, year	Condition/procedure	Prevalence (%)
Jones, 1964	Secondary amenorrhea	1.7
Nawroth et al., 2003	Infertility	6.9
Rochet et al., 1979	Post-caesarean section	2.8
Bergman, 1961	Post-partum D&C (any time)	3.7
Eriksen and Kaestel, 1960	Post-partum D&C (2-4 weeks)	23.4
Adoni et al., 1982	Early spontaneous abortion D&C	6.4
	Late spontaneous abortion D&C	30.9
Schenker, 1982	Missed abortion	35.0
Kralj and Lavric, 1974	Elective abortion D&C*	13.0
Toaff and Ballas, 1978	Recurrent abortion	39.0
Westendorp et al., 1998	Retained products of conception	40.0
Friedler et al., 1993	Spontaneous abortion:	
	One	16.3
	Two	14.0
	Three or more	32.0
Taskin et al., 2000	Hysteroscopic myomectomy:	
	Single	31.3
	Multiple	45.5
	Hysteroscopic metroplasty	6.7

*D&C: dilation and curettage

Ethnicity does not seem to increase the risk of intrauterine adhesions (Dreisler and Kjer, 2019), but elderly women exhibit a higher incidence (Panayotidis et al., 2009). As for technical aspects of hysteroscopic surgery, a higher frequency of IUA has been reported after multiple myomectomy (45%), resection of apposing fibroids (78%), as well as when using monopolar energy (35%), as compared to bipolar energy (7,5%) or (in the case of) preoperative uterine arteries embolization (18-30%) (Gambadauro et al., 2012; Capmas et al., 2013).

### Interest of a prognostic classification

The heterogeneity of conditions leading to IUA and subsequent symptoms results in multiple classifications that are summarized in Table II.

**Table II:** — Different classifications of intrauterine adhesions.

Author, year	Summary of classification
March et al., 1978	Adhesions classified as minimal, moderate, or severe based on hysteroscopic assessment of the degree of uterine cavity involvement
Hamou et al., 1983	Adhesions classified as isthmic, marginal, central, or severe according to hysteroscopic assessment
Valle and Sciarra, 1988	Adhesions classified as mild, moderate, or severe according to hysteroscopic assessment and extent of occlusion (partial or total) at hysterography
Wamsteker, 1990	Complex system classifies IUA* as grade I through IV with several subtypes and incorporates a combination of hysteroscopic and hysterographic findings and clinical symptoms
American Fertility Society, 1988	Complex scored system of mild, moderate, or severe IUA based on extent of endometrial cavity obliteration, appearance of adhesions, and patient menstrual characteristics based on hystero-scopic and hysterographic assessment
Donnez and Nisolle, 1994	Adhesions classified into 6 grades on the basis of location, with postoperative pregnancy rate as primary driver. Hysteroscopy and hysterography are used for assessment
Nasr et al., 2000	Complex system creates a prognostic score by incorporating menstrual and obstetric history with IUA findings at hysteroscopic assessment

*IUA: intrauterine adhesions

A prognostic classification must take into account different symptoms (Table III), as well as findings during evaluation of IUA, including amenorrhea, involvement of the upper uterine cavity, non-visualization of the tubal ostia, post-surgical adhesions, and genital tuberculosis.

**Table III:** — Prognostic classification of intrauterine adhesions.

Class	Symptom	Hysterosalpingography	Appearance of the endometrium on ultrasound
1	Amenorrhea or hypermenorrhea	Adhesions at the level of the internal cervical and/or centrally located adhesions sparing the uterine cavity sidewalls and the fundus	Variable
2	Hypermenorrhea	Adhesions involving the uterine cavity sidewalls including a dysmorphic uterus appearance (t-shaped or clover-leaf)	Regular but usually thin. Normal appearing endometrium in the fundal area that abruptly becomes thin at the midcavitary level
3	Hypermenorrhea	Adhesions involving the upper uterine cavity and the fundus. Unilateral corneal occlusion	Irregular and usually thin
4	Hypermenorrhea	Adhesions involving the upper uterine cavity and the fundus resulting in bilateral corneal occlusion	Irregular and thin
5	Amenorrhea	Total cavity occlusion	Irregular and very thin

### Management of intrauterine adhesions based on a prognostic classification

The preoperative management of IUA consists of hysterosalpingography (HSG) and preoperative estrogen supply during the early follicular phase in menstruating patients, but no use of misoprostol (March, 2011) (Table IV). At the time of surgery, the risk of uterine perforation depends on the type of guidance, where ultrasound guidance has been shown to provide the best results (1.9% of uterine perforations) in comparison to laparoscopic guidance (8.7%) and no guidance (5.3%) (Kresowik et al., 2012).

**Table IV:** — Protocol for managing intrauterine adhesions.

Goals	Means	Protocol
Repair cavity	Scissor lysis under direct visualization	Office HSG and bipolar cutting current
Prevent re-scarring	Intrauterine stent	Intrauterine HA gel
Promote healing	High-dose estradiol	Estradiol
Follow-up		
Architecture	Hysteroscopy or HSG	HSG, mid-cycle 3D USG
Function	Mid-cycle ultrasound of the endometrium	HSG

HSG: hysterosalpingography; HA: hyaluronic acid; USG: ultrasonography.

Adapted from March CM. Management of Asherman’s syndrome. RBMO 2011;23:63-76

The fertility outcome varies according to the prognostic classification (Table V). Of note, after a miscarriage, expectant management is preferable to surgery. A hysterosalpingography must be performed before any surgery to verify any abnormality of the uterine cavity volume and that it has not been decreased by IUA. However, hysterosalpingography has some limitations for this evaluation.

**Table V:** — Fertility outcome and prognostic classification.

	Complete or near complete restoration of the uterine cavity		Partial restoration of the uterine cavity		Minimal or no change in the configuration of the uterine cavity	
	Class 1-2	Class 3-5	Class 1-2	Class 3-5	Class 1-2	Class 3-5
Patients	270	91	11	105	-	56
Desiring pregnancy	270	91	11	101	-	56
Lost to FU	31	9	2	14	-	24
Pregnant	215 (89%)	63 (76.8%)	7 (78%)	38 (43.6%)	-	4 (12.5%)
Delivered	193 (81%)	57 (90.4%)	6 (67%)	29 (76.3%)	-	8 (25%)
Placental complications	6	4	1	8	-	2
Uterine rupture	0	0	1	1	-	1

HSG: hysterosalpingography; HA: hyaluronic acid; USG: ultrasonography.

Adapted from March CM. Management of Asherman’s syndrome. RBMO 2011;23:63-76

## Interauterine adhesions, reproductive outcomes and disease prevention

### 

As previously reported, several procedures can lead to IUA or Asherman syndrome. These terms are often used interchangeably, although Asherman syndrome requires the presence of IUA signs and associated symptoms (pain, menstrual disturbance, subfertility) (Yu et al., 2008).

### What is an adhesiogenic intrauterine surgery?

- Dilation and curettage is recognized as a high adhesiogenic procedure, which has been found to generate incomplete abortion (33.3%), postpartum hemorrhage (37.5%) in further pregnancies (Cenksoy et al., 2013; Li et al., 2019).
- Following caesarean section, the risks of IUA can reach 26.7% after placement of compression sutures for postpartum hemorrhage (PPH) that transverse the uterine cavity (Poujade et al., 2011), whereas uterine compression with U-sutures has been shown to be highly effective and a straightforward emergency procedure which conserves the uterus in these patients (Hackethal et al., 2008). Therefore, this complication was probably underestimated.
- IUA occur in 25.51% of cases after laparotomic myomectomy, strongly indicating that opening of uterine cavity is a risk factor for adhesions (Capmas et al., 2018).
- Is laparoscopic better than laparotomic myomectomy? The results of a non-randomized interventional study showed that the occurrence rate of synechiae in the laparoscopy and laparotomy group was 21% and 19%, respectively (p = 0.99) (Asgari et al., 2015).
- What about operative hysteroscopy? Different studies show that IUA are a major long-term complication of operative hysteroscopy, with a frequency varying from 31% to 45%, according to the pathology initially treated (Taskin et al., 2000; Guida et al., 2004; Mukul and Linn, 2005). At 3 months, the rate was found to be 26.15% (Guida et al., 2004).
- According to a retrospective study, a cold loop resectoscopic myomectomy and diagnostic hysteroscopy two months after surgery seem to be safe and effective procedures associated with a lower rate of intrauterine adhesions (4.2%) (Mazzon et al., 2014).

### IUA after surgical treatment of retained products of conception

A systematic review published in 2016 addressed the issue of long-term complications after the management of retained products of conception (RPOC), which complicate approximately 1% of term-pregnancies (Hooker et al., 2016a) (Table VI). The prevalence of IUA after treatment for RPOC is still undetermined. This systematic review highlighted that, in women treated by D&C, the rate of reported IUA was significantly higher than in women treated by hysteroscopic resection (29.6 % vs. 12.8%, p < 0.0001). Similarly, persistent RPOC were significantly more frequent following D&C than after hysteroscopic resection (28.8% vs. 1.4%, p < 0.0001). These findings suggest that hysteroscopic resection is superior to D&C in women who are suspected of RPOC.

**Table VI:** — Conditions preceding RPOC.

Pregnancy preceding RPOC	Hysteroscopy n= (%)	D&C n= (%)	P value
First and second trimester	23 (15.6%)	10 (5.2%)	< 0.0001
Delivery	71 (48.3%)	140 (72.9%)	< 0.01
Not reported	53 (36.1%)	42 (21.9%)	< 0.01

RPOC: retained products of conception; D&C: dilation and curettage.

Based on: Hooker et al., 2016a.

Regarding the long-term reproductive outcomes, they were similar after D&C and hysteroscopic resection, whereas there was a trend towards earlier conception following hysteroscopic resection. However, data regarding the correlation between the occurrence of adhesion after RPOC treatment and long-term reproductive outcomes are lacking.

### Termination of pregnancy

The termination of pregnancy (TOP) is the most frequent intervention in women worldwide. Approximately one-third of women will resort to TOP at least one time in their reproductive life. In 2016, a systematic review explored the prevalence of IUA after TOP (Hooker et al., 2016b). Only two studies reported the prevalence of IUA after TOP (Table VII) and no study was found regarding medical management only.

**Table VII:** — Prevalence of intrauterine adhesions after termination of pregnancy.

Author	Population	Patients n	System	IUA n (%)
Kajanoja and Aantaa, 1983	Nulliparous women, mid-trimester TOP	173	HSG	28 (16%)
Salat-Baroux, 1984	TOP before 10 weeks of gestation	118	HYS	25 (21%)

Total: 53 (18%)

### Miscarriage

A third systematic review looked for prevalence, risk factors and long-term reproductive outcomes when IUA occurred after a miscarriage (Hooker et al., 2014). Among ten studies of the review, 912 women were included: 86% treated by D&C, 3% medically treated; 2% after spontaneous miscarriage and 9% for whom treatment was not stated. The prevalence of post-miscarriage IUA was 19%, of which 40% of IUA were of moderate to severe extent. Women who experienced ≥ 2 miscarriages have an increased risk of IUA compared to women with one miscarriage (odds ratio: 1.4 to 2.1). The number of D&C procedures seems to be the main risk factor. The data on the correlation between IUA post-miscarriage and long-term reproductive outcome are lacking.

### Disease prevention: hyaluronic acid gel

A multicenter, randomized, blinded, controlled trial analyzed the short-term outcome of women with a history of at least one previous curettage who underwent an application of Auto-cross-linked polysaccharide (ACL)-hyaluronic acid gel after D&C (Hooker et al., 2017). In terms of IUA, the application of ACP-hyaluronic acid gel following a miscarriage showed a significant reduction in the incidence of IUA (13% vs. 30.6% in the control group; RR = 0.43; 95% CI: 0.22-0.83). A significant reduction in the severity of IUA according to the mean adhesion scores (mean difference = - 1.32; range: - 2.00 to - 0.64), and less moderate to severe IUA, according to the American Fertility Society (AFS) classification (RR = 0.09; CI = 0.01-0.6; p = 0.02), as well according to the European Society for Gynecological Endoscopy (ESGE) classification (RR = 0.05; 95CI: 0.006-0.32; p= 0.002).

**Figure 1:**
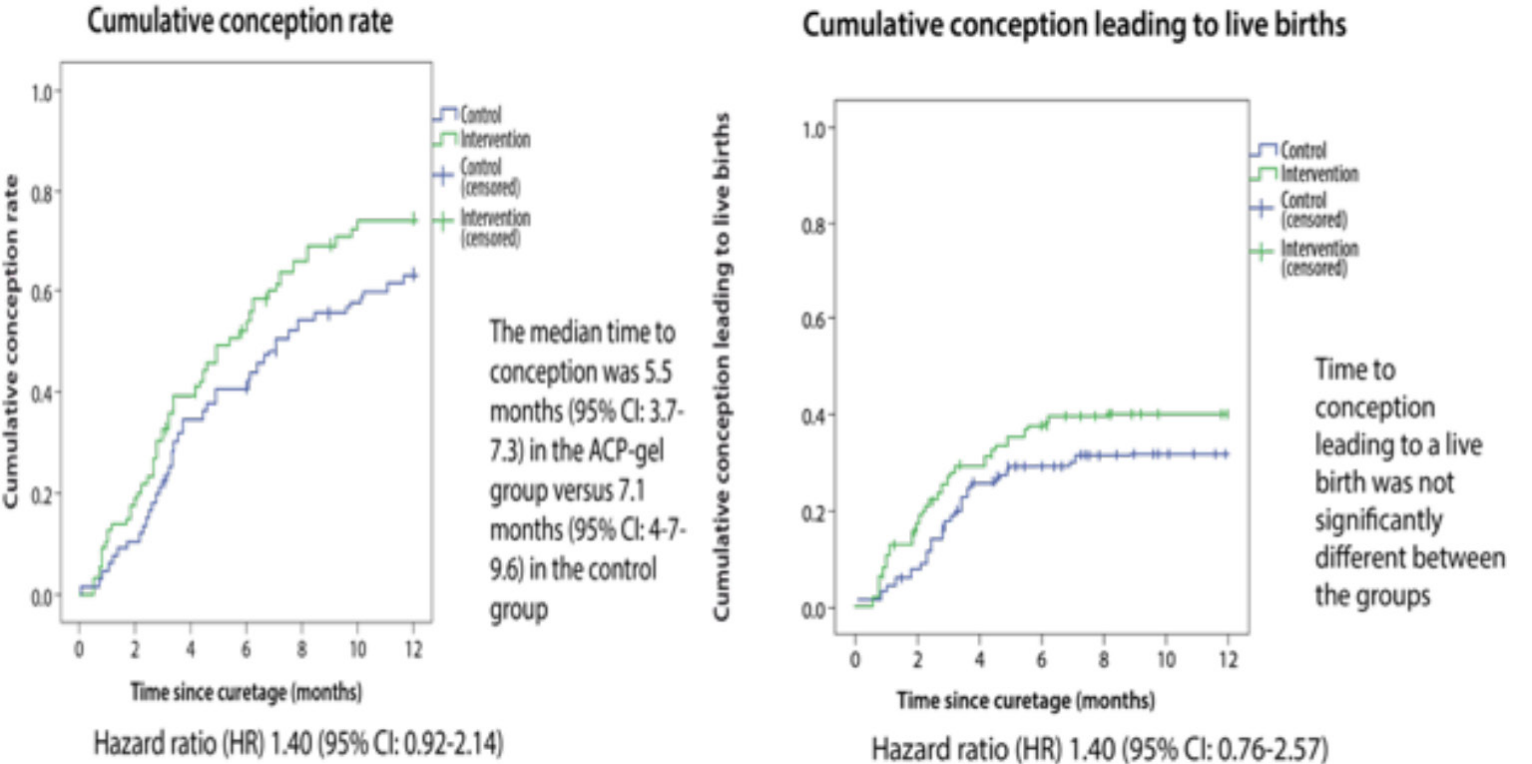
— Reproductive outcomes at 12 months after the use of hyaluronic acid gel. Figures show the conception rates after the application of hyaluronic acid gel following dilatation and curettage in women with, at least, one previous curettage. Reproduced with permission from: Hooker AB, de Leeuw R, van de Ven PM, et al. Prevalence of intrauterine adhesions after the application of hyaluronic acid gel following dilatation and curettage in women with at least one previous curettage: short-term outcomes of a multicenter, prospective randomized controlled trial. Hooker et al., 2017.

In terms of reproductive outcome at 12 months, the use of hyaluronic acid gel after dilatation and curettage showed shorter median time to conception: 5.5 months (95CI: 3.7-7.3) in the hyaluronic acid gel group vs. 7.1 months (95CI: 4.7-9.6) in the control group (Figure 1); (HR = 1.40; 95CI:0.92-2.14). No significant difference between both groups was found regarding time to conception leading to a live birth (HR = 1.40; 95CI: 0.76-2.57) (Figure 1). Noteworthy, a 24-month follow-up is scheduled to further evaluate the reproductive outcomes.

## The evolution of energy expenditure during chemotherapy

The observed changes in REE during cancer treatment are still under debate but more evidence is accumulating that a U-shaped curve, with its nadir during mid-treatment and apex levels at the beginning and end of chemotherapy is found when REE is measured by IC (Figure 1). (Nguyen et al., 2016; Vyas et al., 2014). As mentioned above, literature has revealed that cancer patients during the tumour-bearing state experience an increase in REE of 8-9%. It can be hypothesised that a reduced tumour activity results in a decrease in energy expenditure. Such hypothesis is in line with previous results, where a significant decrease in REE has been reported in patients with complete remission of the tumour after chemotherapy treatment compared to non- or partial responders (Lerebours et al., 1988; Russell et al., 1984). Evidence that is indicative for the decrease in REE, comes from a study on surgical resections. In this study, a decrease in REE was noticed after curative removal of the tumour, and hence, the increasing effects of the tumour on energy expenditure were no longer present (Reeves et al., 2006). Another explanation for the initial decrease can be attributed to weight loss during the first part of the treatment (Juinot et al., 2018). A recent systematic review showed a decrease of approximately 1,5% - 25% in REE can be noticed, depending on tumour type and stage (Figure 1) (unpublished data, Van Soom, personal communication).

The second phase, the incremental section, of the U-shaped curve could be explained by the accumulative effects of chemotherapy due to inflammatory-induced complications (Garcia-Peris et al., 2005). Thus, there is evidence that cancer type, stage and type of chemotherapeutic drugs play an important role during the onset of inflammatory processes in cancer and cancer treatment (Figure 1). (Rusu et al., 2018; Vyas et al., 2014).

## Adhesion prophylaxis after intrauterine surgery

### 

The prevention of IUA has been the subject of several reviews and guidelines (Di Spiezio Sardo et al., 2016; AAGL, 2017; De Wilde et al., 2017). The goals of the surgical strategy should be: avoid trauma to healthy endometrium and myometrium; reduce the use of electrosurgery; avoid forced cervical manipulation. Furthermore, there is an increased likelihood of IUA recurrence after successful adhesiolysis in the case of adhesions originally located at the uterine cornua, the cervico-isthmic region or involving a large portion of the uterine cavity (Yang et al., 2016).

### Which prevention of intrauterine adhesions after surgery?

With regard to type of energy, a French randomized comparative clinical trial was the first to address this issue (Rochet et al., 1979). When it comes to the results of myomectomy, the results showed a significant superiority of bipolar energy over monopolar by reducing the synechiae rate in the resection of type 0-2 fibroids.

Ideal intrauterine anti-adhesive method. The main characteristics of such a method are:

- Effective
- Cheap
- Easy to use
- Acts locally during the main phase of adhesion formation
- Re-absorbable and biocompatible
- No interference with normal tissue repair process

One of the first steps to consider is the kinetic of adhesions formation. IUA development varies from 1 to 4 weeks after endometrial trauma (Rochet et al., 1979; Schenker et al., 1982). Moreover, postoperative new IUA formation is an important factor influencing endometrial wound healing (Yang et al., 2013).

### Pharmacological therapy

The first approach is pharmacologic, even if its role is difficult to evaluate as it is used in combination with other preventive strategies in most studies (Di Spiezio Sardo et al., 2016). A randomized study compared two doses of estrogen (2 mg and 6 mg/day). While this study did not address the fundamental question of whether estrogen adjuvant therapy prevents the recurrence of IUA, the findings did not support the use of high-dose estrogen therapy after hysteroscopic adhesiolysis (Guo et al., 2017). It has been shown that aspirin is able to promote microvascular formation in the endometrium of patients with severe IUA after surgery, and to improve local blood circulation, endometrial growth and repair, and menstruation (Chen et al., 2017).

### Intrauterine devices

The interest of intrauterine devices (IUD) has been also explored. As pharmacological therapy, IUDs are used in combination with other preventive procedures. Data on the efficacy of Foley catheter, intrauterine balloon and amnion graft are limited and mostly derived from non-randomized studies with few patients. Foley catheter (during 7 to 10 days) seems to be more effective than IUD, but less so than intrauterine balloon (Di Spiezio Sardo et al., 2016). Developments on the role of amniotic membrane in humans show that the amnion graft, especially if fresh, seems to improve the efficacy of other barrier methods (Peng et al., 2017).

### Intrauterine gel

Numerous randomized and non-randomized trials have shown that intrauterine application of anti- adhesive gel is an effective strategy to reduce iterative interventions after hysteroscopic surgery due to postoperative IUA (Acunzo et al., 2003; Guida et al., 2004; Di Spiezio Sardo et al., 2011). A reduction of de novo IUA as well as a significant decrease in the severity of IUA has been observed after intrauterine gel application. Intrauterine gel is effective as primary and secondary preventions.

To date, three gels have been developed: auto-crosslinked polysaccharide hyaluronic acid (ACP), hyaluronate carboxymethylcellulose membrane (CH) and polyethylene oxide-sodium carboxymethylcellulose (POC). The association of an early office hysteroscopy (1-2 months later) with miniaturized instruments and the use of an anti-adhesive barrier gel seem to be the best strategy for the prevention and treatment of postsurgical intrauterine and cervical adhesions.

### New approaches

Endometrial reconstruction from stem cells represents a novel cell-based therapy (Gargett and Ye, 2012; Cervelló et al., 2015; Singh et al., 2014). Adult stem-cells can reconstruct endometrial tissue in vivo suggesting their possible use in treating disorders associated with inadequate endometrium. The identification of specific markers for endometrial mesenchymal stem-cells and candidate markers for epithelial progenitor cells enables the potential use of endometrial stem/progenitor cells in reconstructing endometrial tissue in Asherman syndrome and IUA (Garrett and Ye, 2015). One study showed that autologous stem-cell implantation leads to endometrial regeneration reflected by restoration of menstruation in five out of six cases (Singh et al., 2014).

## Prophylaxis in high-risk abdominal surgery and countermeasures

### Clinical impact of adhesions

These lesions can be defined as abnormal fibrous connections which develop between the peritoneum and organs as a result of surgical trauma leading to an imbalance in fibrinolysis (Wallwiener et al., 2014). The surgical risk factors are ischemia, sutures, fibrocoagulation, dry CO_2_ insufflation, high temperature, and exogenous material.

IUA occur in postoperative conditions in about 90% of cases (Menzies and Ellis, 1990), and after 60-90% of procedures (Hirschelmann et al., 2012). Adhesions have an impact on health costs as they require more re-admissions, re-interventions, longer surgical times, longer hospital stays, and more disability (Lower et al., 2004; Wilson, 2007, Herrmann and De Wilde, 2015).

Adhesions occur either after laparoscopy or open surgery. Thereafter, the adhesions limit the use of minimal invasive techniques (van Der Krabben et al., 2000; Swank et al., 2003). The consequences of adhesions are: chronic pelvic and abdominal pains, bowel obstruction; increased risk of miscarriage and premature delivery; complications during surgery such as difficult dissection, and visceral injury (Deans and Abbott, 2014; De Wilde et al., 2016).

### Awareness of adhesions

The main issue is the lack of awareness among surgeons regarding adhesions and their possible consequences. Therefore, the ANGEL survey was conducted to evaluate this topic: the purpose was to ascertain if gynecological surgeons are sufficiently aware of adhesion-related issues and their influence on their practice (Wallwiener et al., 2013). The methodology used was an 18-items questionnaire posted on the ESGE website (8 questions on care setting and surgical practice, 10 questions on adhesions formation and reduction). The analysis included the answers of 414 participants from 36 countries (UK 20.6%, Germany 20%, Italy 16.2%, and Netherlands 7.5%). The majority of participants work at least part-time in a hospital. The importance given to adhesions by participants highlights the need for improvement in patient information (Table VIII).

**Table VIII:** — Results of ANGEL survey: importance given to adhesions.

	Strongly applicable	Applicable	Undetermined	Slightly applicable	Not applicable
Major morbidity	20.2%	50.6%	11.1%	17.0%	1.2%
Regular use of intraoperative agents	22.1%	22.1%	7.9%	17.4%	30.4%
Treatment options for adhesions	14.3%	38.1%	15.6%	18.9%	13.1%
Long-term complication	16.4%	49.2%	11.9%	13.9%	8.6%
Adhesion formation	19.7%	44.7%	13.1%	12.7%	9.8%

**Figure 2:**
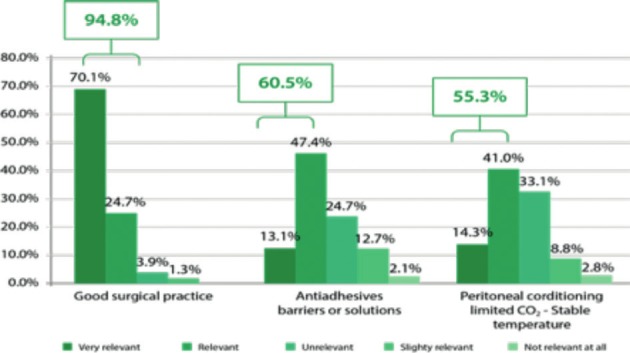
— Good surgical practice and the use of anti-adhesive barriers. Reproduced with permission from: Wallwiener et al., 2013.

The results showed that fewer surgeons expected adhesion formation after laparoscopy (18.9%) than after laparotomy (40.8%). Among a total of 10 mentioned potential adhesion risk factors, the most cited were: abdominal infections, extensive tissue trauma, postoperative infections, and previous surgeries. Endometriosis surgery and myomectomy were recognized to be the most adhesiogenic procedures. Regarding the use of anti-adhesive barriers, they seemed relevant in 60.5% of the cases (Figure 2).

Cumulative evidence and experts in adhesion prophylaxis agree that the reduction in postsurgical adhesion formation is driven by the following:Minimizing peritoneal injury during surgery (i.e. careful surgical technique; gentle tissue handling, microsurgical principles), meticulous hemostasis, the excision of necrotic tissue, minimizing ischemia and desiccation, reducing cautery time, excising tissue rather than coagulating (endometriosis), frequent use of irrigation and aspiration; preventing the introduction of foreign bodies by using non-reactive suture materials, avoiding contamination with surgical glove powder, and the prevention of infection; and placing anti-adhesive barriers between damaged tissues (De Wilde et al., 2017).

### What is considered as good surgical practice and how to reduce postoperative adhesions?

**Figure 3:**
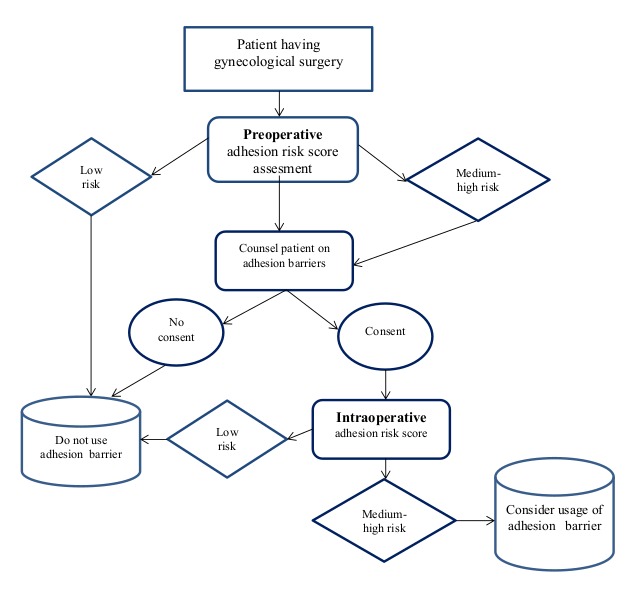
— Strategies for high-risk laparoscopy. Reproduced with permission from: De Wilde et al., 2017.

The strategies for high-risk laparoscopy are presented in Figure 3 (De Wilde et al., 2017). Special considerations are given to agents with data supporting safety in routine surgery and efficacy in adhesion prevention, practicality, ease of use and cost of agents.

### How to prevent adhesions?

**Figure 4:**
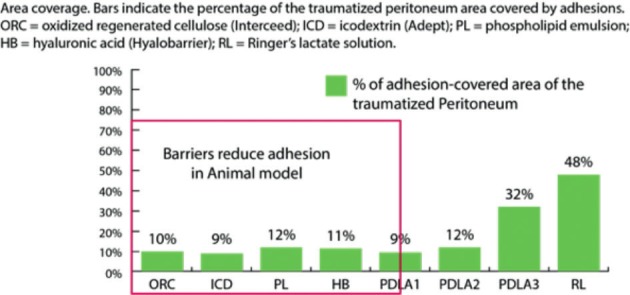
— Anti-adhesive barriers examples. Reproduced with permission from: Wallwiener et al., 2006.

Several anti-adhesive barriers have been developed to prevent the occurrence of adhesions, which have successfully prevented adhesions in animal models (Figure 4) (Wallwiener et al., 2006).

The dextran aldehyde + polyethylene glycolamine polymer (Actamax ® ) showed no serious adverse events possibly device-related in a first in-human study (Trew et al., 2017). It was considered as easy or very easy to use in 54.5% of cases. Some residual material was evident at second-look laparoscopy. The reduction in adhesion score was 41.4% vs. control (p = 0.017), and the reduction reached 49.5% among myomectomy (p=0.008).

The ACP-hyaluronic acid gel (Hyalobarrier ® ) significantly reduced the rate of any IUA and adhesion score following intraperitoneal or intrauterine surgery (Liu et al., 2018). It can prevent IUA, particularly those with moderate severity and a lower adhesion score.

According to a systematic review and meta-analysis, laparoscopic adhesiolysis reduces pain from adhesions in about 70% of patients in the initial phase after treatment (van den Beukel et al., 2017).

Three randomized controlled trials showed the partial success of adhesion prevention in high-risk endometriosis patients by reducing adhesion extent at second-look laparoscopy performed a few weeks after initial surgery (Mais et al., 1995; diZerega et al., 2007; Brown et al., 2007). However, those trials lack data on long-term outcomes such as fertility, pelvic pain, disease recurrences, or other adhesions-related complications. Gels and hydroflotation agents showed to be effective in preventing adhesion during gynecological surgery (Somigliana et al., 2012; Ahmad et al., 2014; Bosteels et al., 2014). There is a lack of evidence concerning an effect of these agents on the improvement of fertility outcomes and postoperative bowel obstruction (ASRM, 2013; Ahmad et al., 2014; Bosteels et al., 2014). Full-conditioning together with the application of a barrier has a potential role in reducing postoperative pain, CRP concentration and facilitating clinical recovery (Koninckx et al., 2013).

### Strategies for high-risk patients

- Raising awareness: counselling patients on adhesions and consequences.
- Indications for patients at risk: consider anti-adhesive barriers in high-risk patients such as those with endometriosis and myomectomies. Full-conditioning plus ACP hyaluronic acid gel barrier could lead to adhesion-free surgery.
- Good surgical practice: minimize trauma. Here, 10% of C02 strongly reduces pain and accelerates recovery, without risks.

## Conclusion

Good practice in both hysteroscopic and abdominal minimally invasive surgery introduced new standards of care aiming to protect the abdominal and uterine cavity from injury during operative procedures. However, the complex physiological peritoneal and endometrial healing mechanisms are not fully understood. There is limited evidence about the efficacy and outcomes of techniques and adjuvant measures used during adhesiolysis. The definition of a universal intrauterine adhesions classification as well as a prognostic scoring system to identify women at high risk of postoperative adhesions are necessary to advise those who could benefit the most of the use of antiadhesion barriers.
